# *Trypanosoma cruzi*-Infected Human Macrophages Shed Proinflammatory Extracellular Vesicles That Enhance Host-Cell Invasion via Toll-Like Receptor 2

**DOI:** 10.3389/fcimb.2020.00099

**Published:** 2020-03-20

**Authors:** André Cronemberger-Andrade, Patrícia Xander, Rodrigo Pedro Soares, Natália Lima Pessoa, Marco Antônio Campos, Cameron C. Ellis, Brian Grajeda, Yifat Ofir-Birin, Igor Correia Almeida, Neta Regev-Rudzki, Ana Claudia Torrecilhas

**Affiliations:** ^1^Departamento de Ciências Farmacêuticas, Universidade Federal de São Paulo (UNIFESP), São Paulo, Brazil; ^2^Instituto René Rachou/FIOCRUZ – MG, Belo Horizonte, Brazil; ^3^Border Biomedical Research Center, Department of Biological Sciences, University of Texas at El Paso (UTEP), El Paso, TX, United States; ^4^Department of Biomolecular Sciences, Weizmann Institute of Science, Rehovot, Israel

**Keywords:** *Trypanosoma cruzi*, toll-like receptor 2, inflammation, extracellular vesicles, macrophage

## Abstract

Extracellular vesicles (EVs) shed by trypomastigote forms of *Trypanosoma cruzi* have the ability to interact with host tissues, increase invasion, and modulate the host innate response. In this study, EVs shed from *T. cruzi or T.cruzi*-infected macrophages were investigated as immunomodulatory agents during the initial steps of infection. Initially, by scanning electron microscopy and nanoparticle tracking analysis, we determined that *T. cruzi*-infected macrophages release higher numbers of EVs (50–300 nm) as compared to non-infected cells. Using Toll-like-receptor 2 (TLR2)-transfected CHO cells, we observed that pre-incubation of these host cells with parasite-derived EVs led to an increase in the percentage of infected cells. In addition, EVs from parasite or *T.cruzi*-infected macrophages or not were able to elicit translocation of NF-κB by interacting with TLR2, and as a consequence, to alter the EVs the gene expression of proinflammatory cytokines (TNF-α, IL-6, and IL-1β), and STAT-1 and STAT-3 signaling pathways. By proteomic analysis, we observed highly significant changes in the protein composition between non-infected and infected host cell-derived EVs. Thus, we observed the potential of EVs derived from *T. cruzi* during infection to maintain the inflammatory response in the host.

## Introduction

Research interest in extracellular vesicles (EVs) and their involvement in cell-cell, cell-pathogen (parasites, viruses, bacteria, and fungi) or pathogen-pathogen communication and modulation processes in infectious and inflammatory diseases has continuously grown in recent years (Campos et al., 2010). The study of EVs has mainly focused on the types of membrane vesicles secreted into the extracellular compartment isolated from different cells, tissues, and biofluids in healthy and pathological conditions. EVs may be divided into exosomes, microvesicles, and apoptotic bodies (Théry et al., 2018; Mathieu et al., 2019; Witwer and Théry, 2019). These types of EVs have different origins, markers, and sizes (Théry et al., 2002; Tkach and Théry, 2016; van Niel et al., 2018; Mathieu et al., 2019; Witwer and Théry, 2019). EVs are heterogeneous membranous particles from 20 nm to 5 μm, differing in their biogenesis, molecular composition, biodistribution, and function (Campos et al., 2010; Lässer et al., 2011; Street et al., 2012; Madison et al., 2014). EVs are secreted by either prokaryotic or eukaryotic cells, thus extending their phenotype (Campos et al., 2010; Torrecilhas et al., 2012).

EVs from parasitic protozoa have been demonstrated as important virulence factors (Gonçalves et al., 1991; Trocoli Torrecilhas et al., 2009; Torrecilhas et al., 2012; Marcilla et al., 2014; Nogueira et al., 2015). They function as cell-to-cell effectors in the host-parasite interaction and manipulation of the host immune system (Aline et al., 2004; Bhatnagar et al., 2007; Pope and Lässer, 2013; Regev-Rudzki et al., 2013; Marcilla et al., 2014; Coakley et al., 2015; Ribeiro et al., 2018). EVs isolated from parasitic protozoa contain a wide variety of molecules, including proteins, glycoconjugates, lipids, RNAs, non-transcribed RNAs, and microRNAs (Torrecilhas et al., 2012). In infection-derived inflammatory processes, EVs can induce release of cytokines and nitric oxide. EVs may promote an inflammatory/immunosuppressive response in the host environment, thus affecting the subsequent immunopathological events, resulting in pathogen escape and invasion of the host cells (Silverman and Reiner, 2011; Nogueira et al., 2015). In infective host cell-derived *T. cruzi* trypomastigotes, α-galactosyl (α-Gal)-enriched EVs strongly trigger proinflammatory responses in murine macrophages via Toll-like receptor (TLR2)-dependent pathway, and the proteomic analysis of these EVs revealed the presence of several members of the TS/gp85 superfamily (Nogueira et al., 2015; Ribeiro et al., 2018). In *Leishmania* spp., it has been shown that promastigote-derived EVs contain virulence factors such as GP63 and lipophosphoglycan (LPG), and several molecules involved in the pathogenesis of *Leishmania* (Silverman et al., 2010; Atayde et al., 2015; Barbosa et al., 2018), suggesting that these EVs can contribute in the leishmaniasis infection and disease progression. Moreover, EVs isolated from *Plasmodium-*infected red blood cells (iRBC) transfer genetic material to the host cell and induce parasite gametocytogenesis, demonstrating a mechanism of interaction and communication with host cells as well as between parasites. In malaria, EVs can also mediate cellular communication processes, delivering virulence factors in the circulation and inflammatory infiltrate (Regev-Rudzki et al., 2013). It is known that inflammatory responses occur during *T. cruzi* infection and that this can be reproduced by the parasite EVs (Nogueira et al., 2015). Likewise, Dr Ramirez's group showed that metacyclic-trypomastigotes induce the release of TGF-β coated plasma membrane vesicles from blood macrophages and lymphocytes, which could further increase parasite infectivity (Cestari et al., 2012; Wyllie and Ramirez, 2017; Gavinho et al., 2018; Rossi et al., 2019). However, it is unknown whether EVs released by *T. cruzi*-infected macrophages or EVs isolated from parasite modulate hotst inflammatory responses and cell invasion.

The invasion of host cells is a key process in *T. cruzi* infection and it has been found that parasite surface molecules play an important role for parasite attachment and entry in the parasitophorous vacuole (Andrews, 2002). Cell-derived trypomastigotes induce lysosome mobilization, cytoskeleton rearrangements, membrane repair and elevation of Ca^2+^ and cAMP concentrations within the host cell (Tardieux et al., 1992, 1994; Burleigh and Andrews, 1998; Andrews, 2002). In fact, these processes are modulated by parasite EVs that were found to contain the major surface glycoconjugates of the parasite (Ribeiro et al., 2018).

Therefore, in this work, we evaluated whether EV shed by *T. cruzi* infected macrophage modulate inflammatory responses and if EVs from cell-derived trypomastigotes could also modify the cell invasion and the signaling mechanisms involved in this process.

## Materials and Methods

### Ethics Statement

All experimental procedures used in this work were approved by the Ethics Committee on Animal Use (CEUA) of the Federal University of São Paulo, protocol 1073090614.

### Cell Lines and Parasite Infection

Tissue culture-derived trypomastigotes (TCT) of the *T. cruzi* Y strain were collected from the culture supernatants after 5 days after infection of LLCMK_2_ epithelial cells (monkey kidney epithelial cell line, ATCC© CCL-7™, VA, USA). Cells were maintained in low-glucose DMEM supplemented with 10% fetal bovine serum (FBS) (Invitrogen, CA, USA) at 37°C in a humidified 5% CO_2_ atmosphere. THP-1 (human peripheral blood monocyte cell line, ATCC© TIB-202™, VA, USA) cells were cultured in RPMI-1640 medium supplemented with 10% FBS and maintained at 37°C in a humidified 5% CO_2_ atmosphere. Cells were tested for *Mycoplasma* contamination by using the polymerase chain reaction (PCR) methodology (Uphoff and Drexler, 2002). The THP-1 monocytes were induced to differentiate into macrophages by the addition of phorbol myristate acetate (50 ng/mL) (Calbiochem, CA, USA) for 24 h in serum-free RPMI 1640. To determine the number of infected cells and the number of intracellular parasites, cells were cultured on circular glass coverslips and subsequently stained with Giemsa.

### Isolation and Characterization of EVs

EVs from trypomastigotes (EV-TY) were obtained by incubation of parasites for 2 h in DMEM containing 2% glucose at 37°C and 5% CO_2_ and purified by size-exclusion chromatography (SEC), as previously described (Ribeiro et al., 2018). To obtain EVs from THP-1 infected and non-infected macrophages (respectively EV-THP-1inf and EV-THP1) were incubated with *T. cruzi* trypomastigotes at a 1:10 ratio (1 mL) for 4 h at 37°C/5% CO_2_ in RPMI 1640 containing 10% ultracentrifuged FBS to remove serum EVs. After infection, the THP-1 macrophages were washed three times with PBS, and then maintained in the same fresh medium for the production of EVs. Controls included THP-1 cells without *T. cruzi* infection. EVs were then recovered at the indicated times by differential ultracentrifugation of culture supernatants. Briefly, the supernatants were submitted to 10 min at 500 × g, followed by 10 min at 3,000 × g and 15 min at 8,000 × g to remove cells and cellular debris. EVs were then pelleted at 100,000 × g for 1 h using a T-890 fixed angle rotor (Thermo Fisher Scientific, MA, USA) as previously described in MISEV guideline (Théry et al., 2018).

The obtained EVs were characterized by NTA analysis to determine concentration and size using NanoSight NS300 (Malvern Instruments, Worcestershire, UK) equipped with a 405 nm laser and coupled to a CCD camera. Data were analyzed using NTA software (version 2.3). Each sample diluted (1:10) in PBS was analyzed in triplicate; and loaded into the instrument for 30 s at 20 frames per second with the camera level set to 14.

### Scanning Electron Microscopy (SEM)

THP-1 cells infected or not with *T. cruzi*, and THP-1 cells treated with EV-TY were fixed in a 2.5% glutaraldehyde solution as reported elsewhere (Nogueira et al., 2015). The cells were post fixed with osmium tetroxide, treated with tannic acid, and dehydrated with ethanol. Samples were observed in a Field Emission FEI Quanta 250 FEG scanning electron microscope (FEI, OR, USA).

### Immunoblotting

EVs samples containing 10 μg of protein, quantified by Micro BCA Assay kit (Thermo Fisher Scientific, MA, USA), were resolved by 12% SDS-PAGE and transferred to nitrocellulose membranes using standard procedures. The presence of exosome proteins and parasite surface proteins was evaluated by incubating membranes with primary antibodies against CD63, CD9, MHC II (Thermo Fisher Scientific, MA, USA) and the serum of a rabbit immunized with the extract of *T.cruzi* TCT membranes (Schenkman et al., 1991). Detection was achieved using horseradish peroxidase-conjugated anti-mouse or anti-rabbit (KPL Antibodies Seracare, MA, USA) secondary Abs and visualized with Pierce ECL Western Blotting Substrate (Thermo Fisher Scientific, MA, USA).

### CHO Cell Lines

The CHO reporter cell lines (CHO/CD14, CHO/CD14/TLR-2, and CHO/CD14/TLR-4) were generated as described (Lien et al., 1999). These reporter cell lines contain a human CD25 gene reporter under the control of the E-selectin promoter, which contains an NF-κB-binding site (Delude et al., 1998; Lien et al., 1999 and Campos et al., 2001). All CHO reporter cell lines were grown in Ham's F-12 medium containing 10% FBS, 10 μg/ml ciprofloxacin, and 400 units/ml hygromycin B. Cells were plated at a concentration of 10^5^/well in 24-well tissue-culture dishes. Cells were incubated with EV-THP-1, EV-THP-1 infected (EV-THP-1 inf) and EV-TY. Medium alone, 100 ng/ml LPS (from *Escherichia coli* serotype 055: B5; Sigma-Aldrich, MI, USA), and UV-killed *Staphylococcus aureus* at a ratio of S. aureus/cell of 500:1 (American Type Culture Collection 12.692), were used as controls. After 24 h of stimulation, cells were stained with PE-labeled anti-CD25 (mouse MAb to human CD25, PE conjugate; Caltag Laboratories, CA, USA). The cells were examined by flow cytometry (BD Biosciences, NJ, USA), and analyses were performed using Cell Quest software (BD Biosciences, NJ, USA) (Campos et al., 2001).

### Infection Assays

TLR2- or TLR4-transfected CHO/CD14 cells were grown on 13 mm diameter glass coverslips in a 24-well dish and washed twice with DMEM/F12 medium (with antibiotics). Cells were incubated with culture supernatant or EV-TY (10^5^ and 10^6^particles/well) for 30 min, at 37°C, in RPMI-10% FBS. These cells were infected with TCTs (1:10, host cell: parasite ratio), for 1 h at 37°C, and then washed twice with RPMI without FBS. Non-adherent parasites were removed by addition of Lymphoprep (Axis-Shield, Norton, MA) to the cell layers, followed by two washes with PBS. The cells were incubated with RPMI-10% FBS for 18 h, at 37°C, or immediately fixed with methanol and stained with Hoechst fluorescent dye (Molecular Probes, Invitrogen Co., Carlsbad, CA). All experiments were performed in triplicate, and 24 photos of each replicate were made using a digital video-imaging fluorescent inverted microscope (Nikon), enabling the counting of infected and non-infected cells.

### Gene Expression

To evaluate the gene expression, quantitative reverse transcriptase polymerase chain reaction (qRT-PCR) was used in THP-1 cells untreated as control, or THP-1 incubated with EV-THP-1 inf, EV-TY (10^8^ particles/mL), or infected with trypomastigotes (TY). Total RNA was obtained from macrophages by using TRIzol reagent (Life Technologies, CA, USA). RNA samples were subjected to DNase treatment and cDNA synthesis with the RevertAid First Strand cDNA Synthesis kit (Thermo Fisher Scientific, MA, USA). RNA samples were quantified by UV absorption in a spectrophotometer (Nanodrop 2000c, Thermo Fisher) followed by electrophoresis in 1.5% agarose gels to assess RNA integrity. RNA samples with high quality and integrity were subjected to DNase treatment (RQ1 RNase-free DNase; Promega, Madison, WI, United States) and cDNA synthesis with the RevertAid First Strand cDNA Synthesis kit (Thermo Fisher Scientific, MA, USA). Specific primers for TLR2, TLR4, IL1β, IL6, TNF-α, STAT1, and STAT3 genes were used to analyze gene expression using RT-qPCR; all sequences were from PrimerBank (https://pga.mgh.harvard.edu/primerbank/) (Supplementary Table 1). The expression levels were normalized to those of the GAPDH and Actin-Beta (ACTB) reference genes using the 2^−ΔΔCt^ cycle threshold method (Schmittgen and Livak, 2008). Differences in the relative expression levels of target genes were determined by com comparison between noninfected cells (as reference samples) and the cells infected or treated with EVs. The gene expression from the reference sample was adjusted to be equal to 1·00. All qRT-PCR procedures were developed following the MIQE guidelines (Bustin et al., 2009).

### LC-MS/MS and Bioinformatic Analysis

EVs (~200 μg of protein) from uninfected and infected macrophages (EV-THP-1 and EV-THP-1inf) were concentrated in a Speedvac. For LC-MS/MS, the filter-aided sample preparation (FASP) method was used, following the manufacturer's protocol (Expedeon, San Diego, CA). The samples were reduced by adding 10 mM DTT for 30 min at room temperature (RT) and centrifuging in a spin filter at 14,000× g for 15 min at rt. After washing twice with 8 M urea in 50 mM Tris-HCl buffer, samples were washed again 2× with the urea/Tris-HCl buffer and 3× with 50 mM ammonium bicarbonate. Then, samples were digested with trypsin (Sigma-Aldrich, St. Louis, MO) was performed, as described by Ribeiro et al. (2018). Peptides were eluted from the filter using 0.1% formic acid and subjected to high-resolution LC-MS/MS analysis in a QE Plus Orbitrap (Thermo Fisher Scientific) equipped with a Nanospray Flex Ion Source (Thermo Fisher Scientific). Using a Dionex UltiMate 3000 RSLCnano UHPLC system (Thermo Fisher Scientific), peptides were separated with a PicoFrit column (75-μm ID × OD 360 μm, 25-cm length, New Objective, Woburn, MA) packed in-house with reversed-phase Aqua C18 porous silica (5 μm, 125 Å, Phenomenex). The column was equilibrated before sample injection at a flow rate of 0.5 μL/min with 95% solvent A (100% H_2_O, 0.1% formic acid) and 5% solvent B (90% acetonitrile, 0.1% formic acid). Samples were then injected onto the C18 column, and the same equilibration phase was run for 10 min. Elution of the peptides was performed using a linear gradient of solvent B up to 35% for 85 min, followed by a 5-min increase to 95%, where the plateau was maintained for 9 min. The column was then re-equilibrated with 5% solvent B for 10 min before injection of the next sample. An automated 2-h run was programmed into the Xcalibur software (Thermo Fisher Scientific), and each sample was analyzed in technical duplicates. Full scan spectra were collected from the 400–1,600 m/z range. Peptides were not excluded based on charge state, and 1 microscan for both full and MS/MS scans were acquired.

The spectra were searched using Proteome Discoverer (PD) 2.1.1.21 (Thermo Fisher Scientific) and filtered via Percolator with an estimated false-discovery rate (FDR) of 1 against sequences from *T. cruzi*, human, bovine, human keratin, and porcine trypsin. Parameters for the database search were as follows: 2 and 1 Da for peptide and fragment mass tolerance, respectively, cysteine carbamidomethylation and methionine oxidation as fixed and variable modifications, respectively. The dataset was further processed through Scaffold Q + 4.8.2 (Proteome Software, Portland, OR), merged, and the probability of their peptide assignments and protein identifications was assessed. Files were exported from Scaffold 4.8.2 for further analysis in Scaffold PerSPECtives (Proteome Software). Only peptides with ≥95% probability were accepted. The criteria for human protein identification included detection of at least two uniquely identified peptides and a probability score of ≥99%. The results were further filtered with the following peptide thresholds: Fisher's exact test (*p*-value) ≤0.001; fold change ≥50% and ≤50%. Gene ontology (GO) annotation was carried out using FunRich (version 3.1.3; http://www.funrich.org/) with the NCBI database (downloaded on 28 March 2019).

### Statistics

All statistical tests were performed using GraphPad Prism version 6 (GraphPad Software, CA, USA). The Mann-Whitney *U*-test was used to determine differences between two groups. Analysis of variance (ANOVA) followed by Tukey's post-test was carried out to compare multiple groups. The differences were considered significant when *p* was < 0.05.

## Results

### Increased Numbers of EVs Are Released From Macrophages Infected With *T. cruzi*

**Figure 1:**
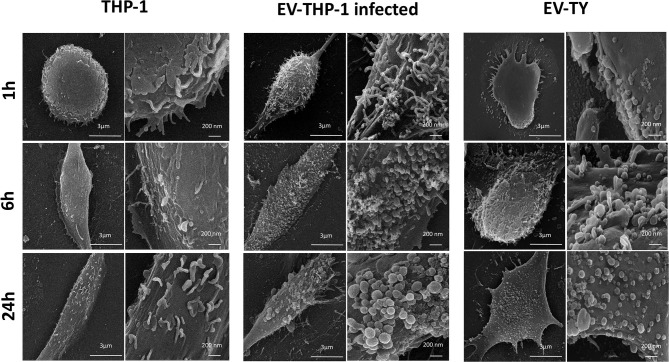
Scanning electron microscopy (SEM) micrographs of cultures of uninfected human macrophages (EV-THP-1), macrophages infected with Y-strain trypomastigotes (EV-THP-1 infected), and macrophages treated with EVs isolated from Y-strain trypomastigotes (EV-TY) for 1, 6, and 24 h. Cells were fixed with glutaraldehyde, and the slides were sent to the Electron Microscopy Center of UNIFESP. The protocol is described in the Materials and Methods section of Nogueira et al. (2015) (8). Images were obtained from macrophages grown on circular glass coverslips and infected with *T. cruzi* (MOI = 10:1) or not, as well as macrophages incubated with Y-strain trypomastigote EVs alone (10^7^ particles/cell).

**Figure 2:**
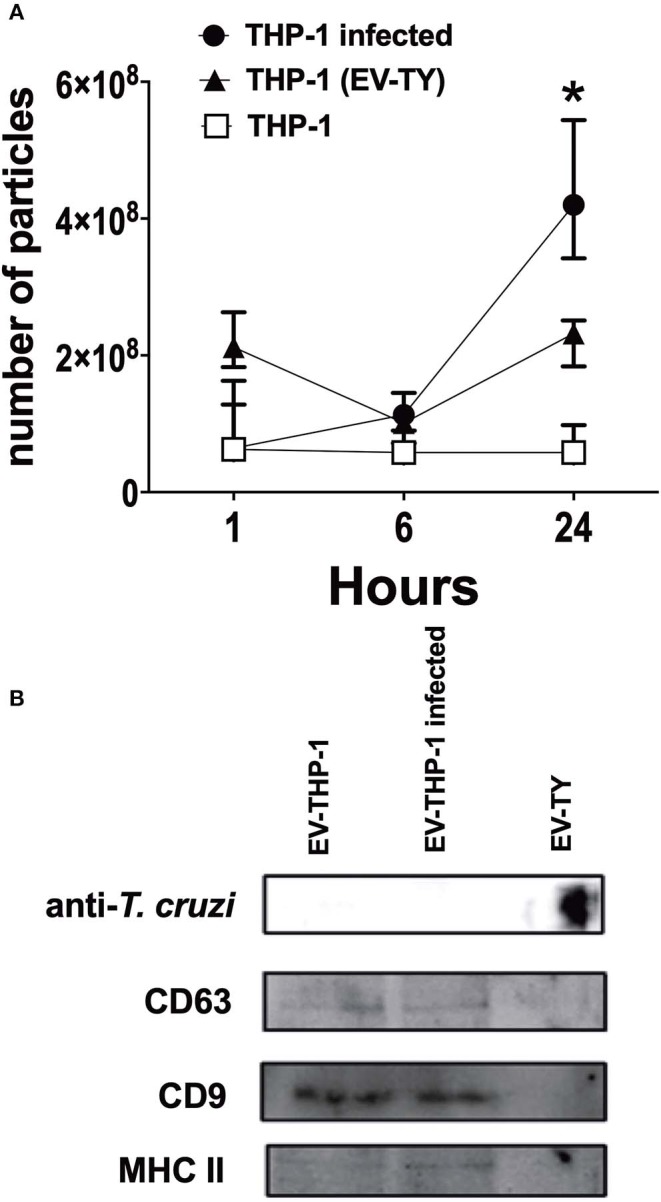
Characterization of EVs isolated from infected macrophages. Kinetics of released of EVs by human THP1 macrophages infected with TCT (EV-THP-1inf) or uninfected (EV-THP-1), or after the addition of *T. cruzi* EVs (EV-TY). Supernatants of macrophages were collected after 1, 6, and 24 h and then EVs isolated by differential ultracentrifugation as described in Methods. **(A)** Particle concentration as measured by NTA. **(B)** Immunoblotting EVs isolated from EV-THP-1inf, EV-THP-1 after 24 h, or EV-TY strain revealed with anti-CD63, anti-CD9, anti-MHC class II and anti-*T. cruzi* polyclonal antibody. Points represent the median of the triplicates, and the dispersion denotes the range. Data are representative of three independent experiments. *Indicates *p* < 0.05 to the respective time of EV-THP-1.

**Figure 3:**
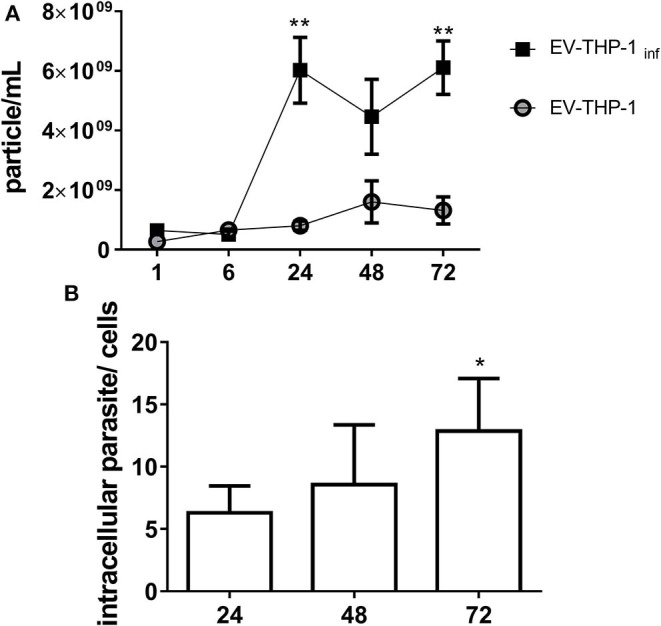
EVs released from THP-1 macrophages during *T. cruzi* infection. **(A)** NTA analysis showing particles/mL EVs released by cells from 1 to 72 h after infection by TCT as described in Methods. *p < 0.01, **p ≤ 0.001. **(B)** Number of intracellular parasites in macrophages at the indicated times as detected by Giemsa staining. The number of intracellular parasites was determined by microscopic examination in 20 fields and at least 200 cells were counted in triplicate samples. The number of intracellular parasites per each cell are shown in the bars with median with range of the triplicates.

Scanning electron microscopy (SEM) micrographs of THP-1-derived macrophages uninfected (EV-THP-1), infected (EV-THP-1 inf), or only treated with EVs from trypomastigotes (EV-TY) showed the presence of round-shaped vesicles with the expected size for exosomes (50–100 nm) and ectosomes (100–200 nm) on the surface of their plasma membrane (Figure 1). Compared to uninfected macrophages and those incubated with EV-TY, infected macrophages released a larger number of vesicles. EVs were isolated from the supernatants of THP-1 macrophages through a series of ultracentrifugation steps. The NTA showed a size distribution of 50–200 nm for all EVs (Supplementary Figure 1). The concentration of vesicles was also measured in the same experiment, revealing an increase in vesicle release by infected THP-1 macrophages (EV-THP-inf) (Figure 2A). In the immunoblotting analysis, the exosomal marker proteins CD63 and CD9 and the surface membrane protein MHC II were detected in EV-THP-1 and EV-THP-1 inf samples (Figure 2B). The presence of parasite surface proteins was only found in the EV-TY group (Figure 2B). The increase in EVs released from infected macrophages is related to presence of parasite from 24 to 72 h. (Figure 3A). However, the EVs release did not appear to increase when the number of parasites augment in infected cells (Figure 3B). After that, cells start to lyse and new trypomastigotes are released in the extracellular milieu.

### The Interaction of EVs With TLR2 Is Critical for the Induction of NF-κB Activation and Increase the Susceptibility of Target Cells to *T. cruzi* Infection

**Figure 4:**
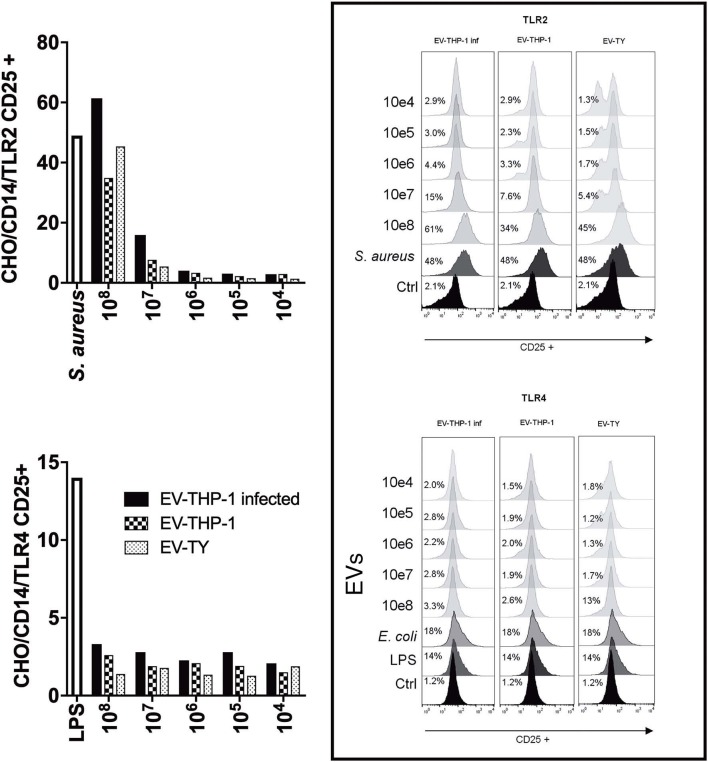
NF-κB translocation assay using CHO reporter cells transfected with TLR2 or TLR4. Flow cytometry of CD25 expression in CHO cells expressing TLR2 or TLR4 and stimulated with EVs derived from infected or non-infected macrophages or with EVs released by the parasite. Fluorescence was analyzed 24 h after stimulation with EVs on a flow cytometer (FACS) (FACScan BD). Bars represent the percentages of positive cells. Data are representative of two independent experiments.

Next, we evaluated the importance of the interaction of EVs from *T. cruzi* trypomastigotes with TLRs for the interaction and internalization of the parasite. The CHO reporter cell line contained an inducible NF-κB-dependent promoter driving the surface expression of CD25 (Campos et al., 2001). CHO cells were incubated with different concentrations of (EV-TY) and with EV-THP-1inf or EV-THP-1. The expression of CD25, which indicates activation of NF-κB, was measured by flow cytometry. These EVs did not increase the induction of CD25 expression by CHO/CD14 or CHO/CD14/TLR4 cells (Figure 4). Previous studies using wild-type, TLR2- or TLR4-knockout murine macrophages from C57BL/6 mice (WT, TLR2^−/−^, or TLR4^−/−^ C57BL/6) showed that TLR2 but not in TLR4 was required for production of nitric oxide (NO) or TNF-α following stimulation with EV-TY and activate MAPKs (ERK 1/2, p38 and JNK) (Nogueira et al., 2015). The data presented in Figure 4 confirmed these previous results showing that the expression of CD25 through the activation of NF-κB was induced in CHO/CD14/TLR2, but not CHO/CD14/TLR4, exposed to EV-TY. Importantly, EV-THP-1 and EV-THP-1inf were also found to quantitatively promote the expression of CD25 in CHO/CD14/TLR2 cells, but not TLR4 (Figure 4), indicating that both EVs derived from *T. cruzi* (EV-TY) and macrophages interact with TLR2, but not TLR4, activating NF-κB. This result also suggested that induction by macrophage EVs was not due to the presence of parasite molecules, as when we used the same number of particles of non-infected macrophages, the induction was similar. The presence of tGPI-mucins and glycoinositolphospholipids (GIPLs) in the EV-TY was likely responsible for the induction of potent inflammatory responses in macrophages (Almeida et al., 2000; Trocoli Torrecilhas et al., 2009).

**Figure 5:**
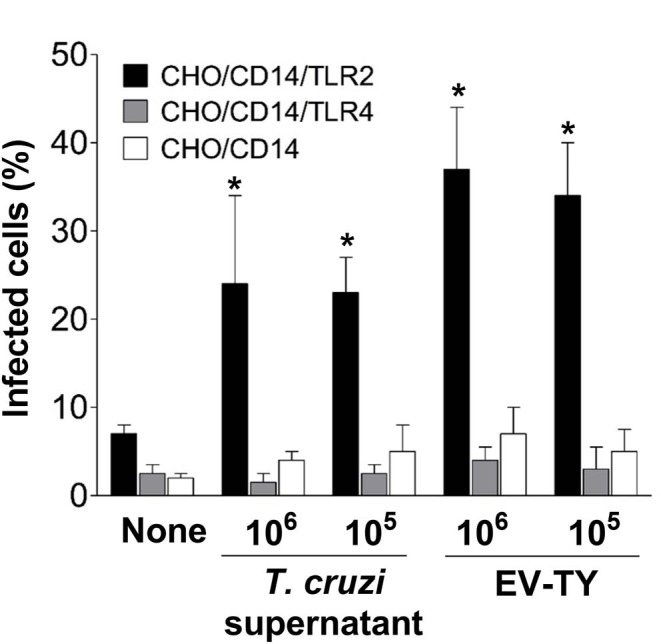
Invasion of TLR2- and TLR4-transfected CHO/CD14 cells with trypomastigotes in the presence or absence of trypomastigotes culture supernatant and EVs isolated from the *T. cruzi* Y strain. Macrophages were preincubated with EVs for 30 min at 37°C in RPMI medium without FCS. Cells were washed with PBS (3×) and Lymphoprep (1×) and then infected with trypomastigotes at a 1:10 ratio (host cell:parasite) for 2 h at 37°C. Cells were then washed (3×) with PBS to remove non-adherent parasites and incubated in RPMI medium with 10% FCS for 24 h at 37°C. Cells were fixed with 100% methanol. Then, the cells were stained with 4′,6-diamidino-2-phenylindole (DAPI). The number of infected cells was estimated using an inverted Nikon fluorescence microscope. *p < 0.01.

The participation of TLR2 in the host-cell infection by *T. cruzi* was then investigated. To test this hypothesis, infection experiments were performed in CHO/CD14 cells, transfected or not with TLR2 or TLR4. Upon prior exposure to culture supernatants of trypomastigotes or EVs, cells transfected with TLR2, but not TLR4- or non-transfected cells were increase the susceptibility of target cells to *T. cruzi* infection. A remarkable (up to 6–7-fold) increase in percentage of infected cells was observed in TLR2-transfected CHO/CD14 cells exposed either to total shedding or EV-TY (Figure 5).

### EVs Induce the Differential Expression of TLR2, TLR4, IL-1β, IL-6, TNF-α and STAT1/STAT3

**Figure 6:**
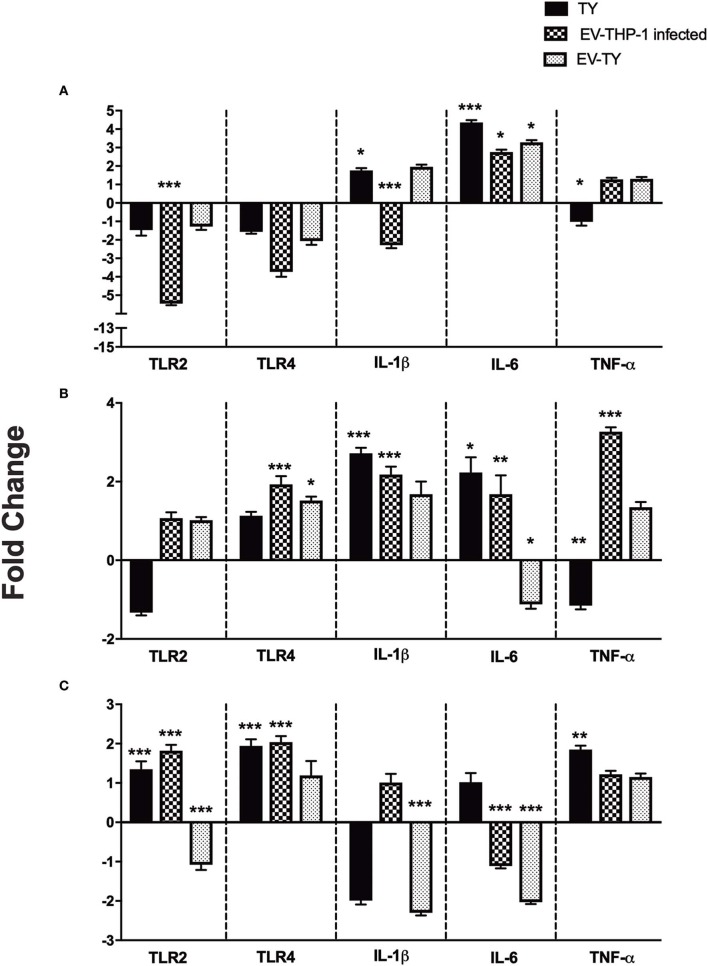
Gene expression levels of TLR2, TLR4, IL-1β, IL-6, and TNF-α proinflammatory cytokines in differentiated THP-1 cells. Relative mRNA expression of TLRs and cytokines were measured after 1 **(A)**, 6 **(B)**, and 24 h **(C)** of macrophages infected with 5 × 10^7^/mL trypomastigotes (TY), or macrophages treated with EV-THP1 inf, EV-TY (both EVs at 10^8^ particles/mL). After these treatments, RNA was extracted, and the expression of TLR2, TLR4 and specific cytokines was determined by RT-qPCR. Bars represent the mean of the triplicates, and the dispersion denotes the standard deviation subtracted to THP-1 macrophages maintained in medium. Data are representative of two independent experiments. Statistical analysis with ANOVA, compared to the respective control; *p < 0.05; **p < 0.01; and ***p < 0.001.

TLRs are involved in the recognition of *T. cruzi* following the activation of host cells to produce TNF-α, IL-12, and NO (Campos et al., 2004; Koga et al., 2006). Macrophages showed differences in TLR gene expression upon stimulation with EVs and *T. cruzi* infection. We analyzed the gene expression of TLR2, TLR4 by RT-qPCR in macrophages untreated and treated with EV-TY, EV-THP-1control, and EV-THP-1 inf. The gene expression analysis in macrophages treated with EV-THP-1inf and EV-TY revealed alterations in TLR2 and TLR4 (Figure 6).

TLR2 expression is essential for proinflammatory cytokine production (Campos and Gazzinelli, 2004). RT-qPCR analysis was performed to assess the changes in gene expression of cytokines and transcription factors. Macrophages, therefore, were incubated with EVs (EV-THP-1 control, EV-THP-1 inf and EV-TY) and also infected with *T. cruzi*. Evaluation of the cytokine gene expression profile revealed an increase in IL-1β levels after 6 h in both *T. cruzi*-infected cells and in those exposed to EV-THP-1inf (Figure 6). There was also an increase in the transcript levels of IL-1β after 1 h of *T. cruzi* infection. After 24 h of *T. cruzi* infection or stimulation with EV-THP-1 inf, no change in IL-1β transcript levels was observed, whereas a marked reduction was seen in cells that were treated with EV-TY or EV-THP-1. After 1 and 6 h of *T. cruzi* infection, the cells showed elevated transcript levels of IL-6. The same result was observed after 1 and 6 h of treatment with EV-TY or EV-THP-1 inf. However, 24 h after the other treatments, there was a reduction in IL-6 gene expression, except for *T. cruzi* infection. In the TNF-α gene expression analysis, elevation of the transcript level was observed after 1 and 6 h of treatment with EV-THP-1inf and after 6 h of treatment with EV-THP-1. Only *T. cruzi* infection induced the gene expression of TNF-α at 24 h.

**Figure 7:**
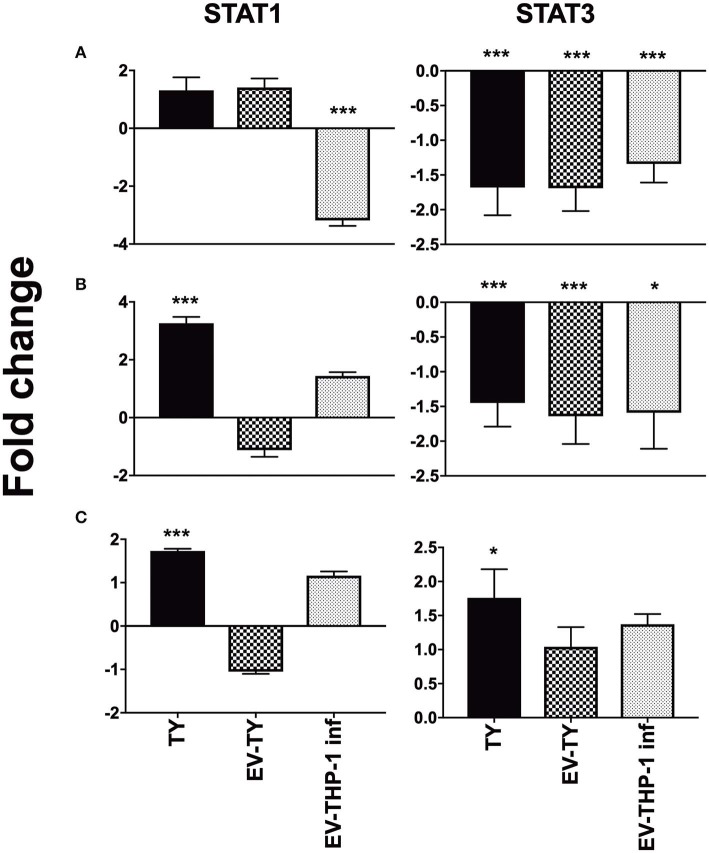
Gene expression of STAT1 and STAT3 in differentiated THP-1 cells. Relative mRNA expression levels of STAT1 and STAT3 transcription factors in macrophages after 1 **(A)**, 6 **(B)**, and 24 h **(C)** of macrophages infected with 5 × 10^7^/mL trypomastigotes (TY), or macrophages treated with EV-THP1 inf, EV-TY (both EVs at 10^8^ particles/mL). After these treatments, RNA was extracted, and the expression of TLR2, TLR4 and specific cytokines was determined by RT-qPCR. Bars represent the mean of the triplicates, and the dispersion denotes the standard deviation subtracted to THP-1 macrophages maintained in medium. Data are representative of two independent experiments. Statistical analysis with ANOVA, compared to the respective control; *p < 0.05; **p < 0.01; ***p < 0.001.

Signal transduction through cytokine receptor-binding occurs via the JAK/STAT pathway signaling. We observed the gene expression of STAT1 and STAT3 in the macrophages stimulated with the different EVs. STAT1 and STAT3 gene expression increased only in response to *T. cruzi* infection (Figure 7). Stimulation with the different EVs reduced the gene expression of STAT3 at 1 and 6 h (Figure 7).

### EVs Released by Infected Macrophages Have Distinct Contents and Parasite Proteins

**Figure 8:**
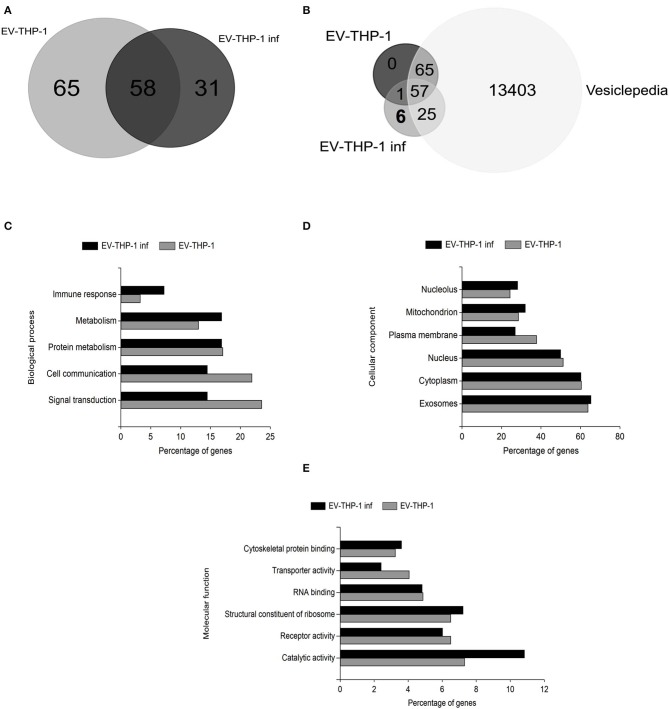
Proteomic and gene ontology analysis of EVs derived from infected or non-infected macrophages. Common and unique proteins identified by LC-MS/MS in EVs from infected or non-infected macrophages **(A)**. Comparison of identified proteins by LC-MS/MS in EVs from infected or non-infected macrophages, *T. cruzi* and Exocarta database proteins (Vesiclepedia) **(B)**. GO graphs of the proteins identified in EV-THP-1 and EV-THP-1inf indicating changes in biological process and molecular functions **(C–E)**.

We performed mass spectrometry-based proteomic analysis (LC-MS/MS) to determine the content of the EVs purified from uninfected (EV-THP-1) and infected macrophages (EV-THP-1 inf). A total of 123 proteins were found in THP-1-EVs and 89 were found in EV-THP-1 inf, from a total of 154 proteins in both samples (Figure 8A). Using FunRich (version 3.1.3), we compared the list of identified proteins to previously published EV data in the Exocarta database (www.exocarta.org) and found that the majority of the proteins had been previously observed to be present in EVs from different sources (Figure 8B). *T. cruzi*-infected host-cell derived EVs carry proteins from the parasite, as previously described (Ramirez et al., 2017). We also looked for *T. cruzi* proteins in EV-THP-1 inf. We found 6 proteins (i.e., HSP60, tryparedoxin peroxidase, trans-sialidase Group II, flagellar calcium-binding protein, surface protein TolT and paraflagellar rod protein 2) (Supplementary Table 1). We also compared the levels of abundance of the 58 common proteins by calculating their fold change. The abundance of 20 proteins increased and 38 decreased after infection with *T. cruzi* (Supplementary Table 2). Most of the proteins found in EV-THP-1 and EV-THP-1 inf are involved in binding, immunological and metabolic processes based on GO term annotations (Figure 8C). Moreover, the majority of the proteins in EV-THP-1 and EV-THP-1 inf were localized in exosomes (Figure 8D). Noteworthy, there was a higher percentage of proteins with catalytic activity in EV-THP-1 inf, particularly enzymes such as trans-sialidase (Figure 8E). It is possible that some of these proteins from *T. cruzi* were not directly associated with the EVs obtained from infected macrophages and were released in the culture supernatant of the infected cells concomitantly with EV-THP-1 inf production. Furthermore, although unlike, the presence of remaining proteins from the parasites used to infected cells cannot be fully excluded.

## Discussion

Macrophages and other mononuclear cells are the host's first line of defense to combat infection of intracellular parasites, such as *T. cruzi*. The release of EVs by *T. cruzi* promotes the activation of murine macrophages (Nogueira et al., 2015). Here, we demonstrate that EVs derived from macrophages infected with *T. cruzi* also modulate the activation of other human THP-1 macrophages to maintain the inflammatory response in the course of infection.

The recognition and response to pathogenic organisms by the immune system is essential for infection control. The expression of TLRs is critical for the response during the early stages of infection for the activation of the immune response. A large number of pathogens express ligands for different TLRs. *T. cruzi* trypomastigote-derived mucin-like glycoproteins (tGPI-mucins) are strong proinflammatory molecules and agonists for the TLR2-TLR6 heterodimer (Campos et al., 2001). *T. cruzi* also express other glycoconjugates such as GIPLs, which activate TLR4 (Almeida et al., 2000; Oliveira et al., 2004).

In recent years, there has been a growing number of studies showing the capacity of EVs to modulate the immune response, especially in the relationship between parasite and host (Campos et al., 2010; Marcilla et al., 2014). Different strains of *T. cruzi* have been shown to release EVs and promote the activation of macrophages via TLR2 (Nogueira et al., 2015). EVs isolated from macrophages infected with intracellular pathogens (*Mycobacterium bovis* BCG and *M. tuberculosis*) induce a TLR/MyD88-dependent proinflammatory response in naive macrophages (Bhatnagar et al., 2007). In this work, we observed that EVs derived from *T. cruzi-*infected THP-1 human macrophages were able to induce the translocation of NF-κB to the nucleus by interacting with TLR2. EVs released by uninfected THP-1 cells also activated the cells via TLR2. However, the amount of EVs produced by the infected cells was 10-fold higher than that produced by the uninfected cells, showing that the amount of EVs is the prime factor for activation. In addition, uninfected macrophages produce EVs when there is tissue damage, which leads to the activation of these cells or dendritic cells and results in the secretion of TNF-α and other inflammatory mediators through the activation of the p38 MAPK pathway and NF-κB (Thomas and Salter, 2010). Damage-associated molecular pattern molecules (DAMPs) could also be related to the mechanism of action of the EVs described herein. Among the DAMPs, we found heat shock proteins, hyaluronic acid, β-defensin 3 and HMGB1, which are recognized by TLR2 (Scheibner et al., 2006; Funderburg et al., 2007; Curtin et al., 2009).

Internalization and phagocytosis by phagocytic cells such as macrophages are crucial processes for initiation of the killing of intracellular pathogens. *T. cruzi* can invade different mammalian cell types (Yoshida, 2006). It has been demonstrated that metacyclic trypomastigotes can invade cells in a Ca^2+^-dependent manner by exocytosis and the recruitment of host lysosomes to the plasma membrane (Tardieux et al., 1994; Rodríguez et al., 1995). Another mechanism for *T. cruzi* entry in cells independent of lysosome recruitment has been described to occur through the activation of PI3K (Woolsey et al., 2003). Here we observed that macrophages infected with *T. cruzi*, as evaluated by SEM, showed an increase in the number of EVs budding from the plasma membrane after the 24-h culture period. This increase was also confirmed by NTA. The release of EVs can be regulated by changes in intracellular calcium levels. When cells were treated with calcium ionophore (A23187), an increase in the release of EVs was found (Savina et al., 2003). *T. cruzi* infection increases the Ca^2+^ concentration in macrophages (Wilkowsky et al., 1996). Additionally, activated cells release a greater number of particles than non-activated cells (Théry et al., 2009). Thus, the increased release of EVs by infected macrophages is related to an increase in intracellular calcium after *T. cruzi* infection and macrophage activation. The size range of the EVs isolated herein (50–200 nm) corroborates that described in the literature (Raposo and Stoorvogel, 2013; Wang et al., 2015; Hui et al., 2018). EVs can further enhance the parasite's ability to invade the host cells through TLR2 signaling. We observed an increase in the number of infected cells when they expressed TLR2 and were incubated with parasite-derived EVs (EV-TY). EVs from *T. cruzi* participate in invasion through a mechanism independent of lysosome recruitment. The parasite invasion is associated with activation of PI3K (Wilkowsky et al., 2001). TLR2 activates PI3K during *T. cruzi* infection, leading to Rab5 activation, which is essential for phagosome formation (Maganto-Garcia et al., 2008). The release of EVs by infected macrophages is related to increased parasite replication, which was observed in macrophages infected with *T. cruzi* herein.

Similarly, in mice infected with *Mycobacterium bovis*, an increase in the release of plasma EVs is also related to an increase in the bacterial load (Singh et al., 2012).

We performed proteomic analysis to compare the content of the EVs released from uninfected THP-1 cells and THP-1 cells infected with *T. cruzi*. According to the Exocarta database (www.exocarta.org), all the proteins found in the EVs have previously been identified in different types of cells and body fluids. The infection changed the composition of the EVs from the host cells; 65 proteins were unique to the EV-THP-1 cells, 31 were unique to the EV-THP-1 inf, and the expression of 58 proteins was altered in both EVs (20 proteins increase and 38 decrease).

The EVs isolated from macrophage supernatants showed the presence of the MHC II surface marker and the exosomal markers CD9 and CD63. No *T. cruzi* antigens could be detected by western blotting in EV-THP-1 and the presence of plasma membrane glycoproteins expressing α-Gal epitopes (i.e., TcMUCII mucins or tGPI-mucins) of the parasite in the EVs of macrophages infected by *T. cruzi* were not identified by total proteomic analysis. In this analysis only six parasite proteins were found in EVs from macrophages infected with *T. cruzi* i.e., HSP60, tryparedoxin peroxidase, trans-sialidase Group II, flagellar calcium-binding protein, surface protein TolT and paraflagellar rod protein 2 (Lobo et al., 2019). These parasite proteins are involved in the recognition of the parasite by host cells, invasion, the adhesion of host cells, metabolism and the induction of the host immune response (Godsel et al., 1995; Quanquin et al., 1999; Mattos et al., 2014; Urményi et al., 2014; Girard et al., 2018).

Previous studies of EVs derived from macrophages infected with intracellular microorganisms, such as mycobacteria and *Leishmania*, also identified proteins of these intracellular organisms that can be carried by EVs (Giri et al., 2010; Hassani and Olivier, 2013).

A variation in cytokine expression in macrophages stimulated with EVs was observed by RT-qPCR. An increase in IL-1β expression was evidenced in cells stimulated with TY and EV-THP-1inf at 6 and 24 h. IL-1β is related to the resistance of the host to infection through stimulation of the IL-1 receptor and activation of the adapter protein MyD88, leading to the production of NO in macrophages (Lima-Junior et al., 2013). Thus, increased IL-1β production may be important in infection control. In addition, immune activation through TLR2 and NF-κB receptors by macrophages has been found to rapidly elevate IL-1β levels (Petersen et al., 2005). Macrophages stimulated with EV-THP-1 and EV-THP-1 inf as well as those infected with *T. cruzi* displayed increased TNF-α expression. TNF-α is a proinflammatory cytokine produced by macrophages and lymphocytes and is important for the control of *T. cruzi* infection. The production of TNF-α by macrophages when stimulated with INF-γ is associated with resistance to infection in mice (Silva et al., 1995). Increased IL-6 production was seen only at 6 h in response to treatment with the EVs of infected macrophages and parasites. Interleukin-6 is a proinflammatory cytokine produced primarily by T cells, dendritic cells and macrophages. It has been reported that IL-6 is not required for a strong Th1 response (Moskowitz et al., 1997) and that in the presence of TGF-β, IL-1β, and IL-23 cause the differentiation of Th0 lymphocytes into Th17 lymphocytes (Acosta-Rodriguez et al., 2007; Zhou et al., 2007).

The cytokine expression profile observed after stimulation with EVs released by infected cells demonstrates the modulation of the proinflammatory conditions caused by the release of these EVs. This cytokine expression profile also occurs in response to EVs from *Leishmania amazonensis*-infected macrophages, which promote the production of the inflammatory cytokines IL-12, IL-1β, and TNF-α (Cronemberger-Andrade et al., 2014). The production of TNF-α, IL-1β and IL-6 by macrophages is associated with the M1 type phenotype. M1 macrophages are responsible for polarizing the Th1 immune response, producing IL-12 and maintaining a low production level of IL-10. The main stimuli that favor the activation and polarization in M1 macrophages are LPS and IFN-γ (Mantovani et al., 2004). It has recently been demonstrated that plasma-isolated EVs from chronic Chagas disease patients promote increased regulation of genes related to the proinflammatory response in macrophages (Chowdhury et al., 2017). In addition, the composition of the EVs isolated from the plasma of mice and patients revealed them to be of cardiac, lymphocyte, and macrophage origin (Chowdhury et al., 2017) thus demonstrating the importance of the potential modulation and activation of macrophages in the chronic phase of Chagas disease.

Signal transduction through IFN-γ receptors is mediated by the phosphorylation of JAK and STAT1. The activation of STAT1 is important for the production of a proinflammatory response. In STAT1 knockout mice, an increase in mortality was found due to increased numbers of parasites found in the blood and tissues (Kulkarni et al., 2015). We did not observe regulation by this mechanism after stimulation with the different EVs. STAT3 phosphorylation induces transcription of the gene encoding SOCS-3 (He et al., 2003). In macrophages infected with *M. tuberculosis*, NF-κB activation was found to be inhibited, and SOCS-3 expression was found to be induced as a mechanism of inflammatory response evasion (Nair et al., 2011; Hillmer et al., 2016). The production of IL-10 leads to increased expression of SOCS-3 and STAT3 in *T. cruzi*-infected cardiomyocytes due to the inactivation of NF-κB and ERK/MAPK (Hovsepian et al., 2013). In the analysis of STAT3 expression, we observed a reduction in expression at 1 and 6 h and an increase only at 24 h after *T. cruzi* infection. The EVs released by infected or non-infected macrophages, as well as the parasite-derived EVs, modulated the response of macrophages favoring the maintenance of a proinflammatory response. It was previously shown that *T. cruzi* GIPLs were not able to activate STAT1 or STAT3 (Stahl et al., 2013). However, in that study the authors did not evaluate tGPI-mucins, which have much stronger proinflammatory than GIPLs (Almeida et al., 2000; Campos et al., 2001). Therefore, we conclude that tGPI-mucins (or TcMUCII mucins), which are present in trypomastigote-derived EVs (Ribeiro et al., 2018), and/or other molecules expressed on the surface of the parasite and the EVs (EV-TY) there from -TY may be involved in the activation of these transcription factors.

In summary, our results indicate that signaling through EVs during *T. cruzi* infection is essential in host-parasite interactions. After infection, increased amounts of EVs are released from infected macrophages that interact with TLR2 and stimulate the translocation of NF-κB. As a result of this interaction and activation, proinflammatory cytokines (TNF-α, IL-6, and IL-1β) are produced and maintain the inflammatory response generated by *T. cruzi* infection. In addition, human macrophage-derived EVs carry parasite proteins, which could be the reason for their increased ability to induce inflammatory responses.

## Data Availability Statement

All datasets generated for this study are included in the article/Supplementary Material.

## Ethics Statement

All experimental procedures used in this work were approved by the Ethics Committee on Animal Use (CEUA) of the Federal University of São Paulo, protocol 1073090614.

## Author Contributions

AC-A, PX, IA, YO-B, NR-R, and AT conceived and designed the experiments. AC-A, PX, YO-B, and AT performed most experiments. NP and MC assisted in NF-κB translocation assay. IA, CE, and BG performed the proteomic analysis. AC-A, PX, YO-B, and AT wrote the manuscript. AC-A, PX, RS, YO-B, IA, NR-R, and AT contributed to final manuscript. All the authors reviewed the manuscript.

### Conflict of Interest

The authors declare that the research was conducted in the absence of any commercial or financial relationships that could be construed as a potential conflict of interest.

## Supplementary Material

Supplementary Figure 1 Size distribution of EVs from macrophages. Kinetics of released EVs isolated from THP-1 cells (differentiated to macrophages) infected or uninfected with *T. cruzi* and or treated with *T. cruzi* EVs. Supernatants from infected or not macrophages and from macrophages incubated with *T. cruzi* EVs were collected after 1, 6, and 24 h and then isolated by ultracentrifugation.

Supplementary Table 1 Proteins identified by LC-MS/MS in EVs derived from uninfected (THP-1) and infected (THP-1 infected) macrophages.

Supplementary Table 2 Table containing sequences of the primers selected for the gene expression analysis of human macrophages stimulated with EVs derived from infected or non-infected macrophages and infected with *T. cruzi* strain Y trypomastigotes.
