# Supplementary figures and images for: Trypanosoma cruzi-Infected Human Macrophages Shed Proinflammatory Extracellular Vesicles That Enhance Host-Cell Invasion via Toll-Like Receptor 2
Source: Front Cell Infect Microbiol. 2020 Mar 20;10:99. doi: 10.3389/fcimb.2020.00099 (PMC7098991; doi:10.3389/fcimb.2020.00099)

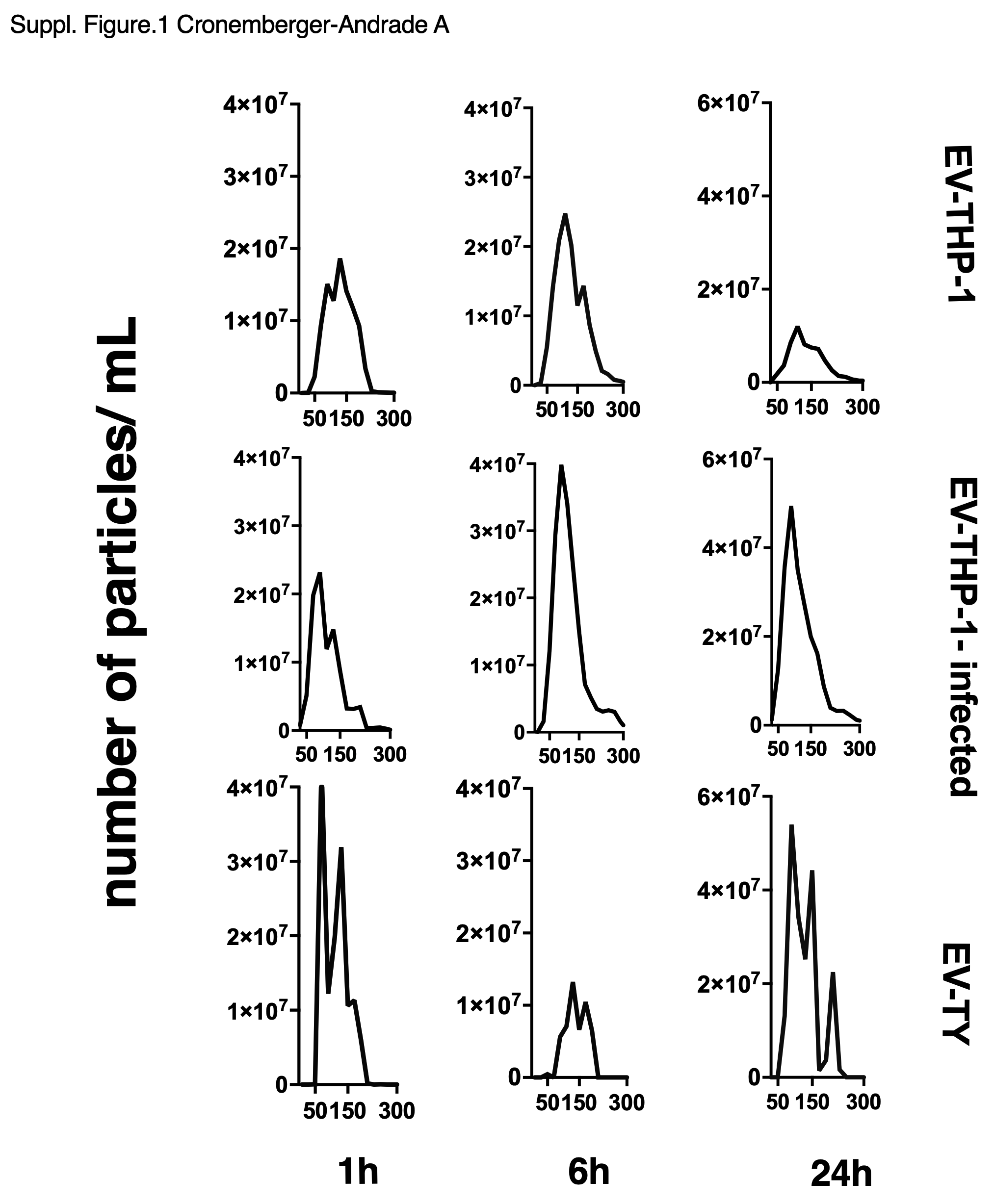

Supplement: Supplementary Figure 1 — Size distribution of EVs from macrophages. Kinetics of released EVs isolated from THP-1 cells (differentiated to macrophages) infected or uninfected with T. cruzi and or treated with T. cruzi EVs. Supernatants from infected or not macrophages and from macrophages incubated with T. cruzi EVs were collected after 1, 6, and 24 h and then isolated by ultracentrifugation. [file Image_1.TIFF]
