# Supplementary material for: Trypanosoma cruzi-Infected Human Macrophages Shed Proinflammatory Extracellular Vesicles That Enhance Host-Cell Invasion via Toll-Like Receptor 2
Source: Front Cell Infect Microbiol. 2020 Mar 20;10:99. doi: 10.3389/fcimb.2020.00099 (PMC7098991; doi:10.3389/fcimb.2020.00099)
Supplement: Supplementary Table 1 — Proteins identified by LC-MS/MS in EVs derived from uninfected (THP-1) and infected (THP-1 infected) macrophages. [file Table_1.docx]

Suppl. Table 1 Cronemberger-Andrade A

| Primers | | Sequence |
| --- | --- | --- |
| TLR2 | **Forward** | ATCCTCCAATCAGGCTTCTCT |
|  | **Reverse** | GGACAGGTCAAGGCTTTTTACA |
| TLR4 | **Forward** | AGACCTGTCCCTGAACCCTAT |
|  | **Reverse** | CGATGGACTTCTAAACCAGCCA |
| STAT3 | **Forward** | CAGCAGCTTGACACACGGTA |
|  | **Reverse** | AAACACCAAAGTGGCATGTGA |
| STAT6 | **Forward** | GTTCCGCCACTTGCCAATG |
|  | **Reverse** | TGGATCTCCCCTACTCGGTG |
| TNF-α | **Forward** | CCTCTCTCTAATCAGCCCTCTG |
|  | **Reverse** | GAGGACCTGGGAGTAGATGAG |
| IL1β | **Forward** | ATGATGGCTTATTACAGTGGCAA |
|  | **Reverse** | GTCGGAGATTCGTAGCTGGA |
| IL-6 | **Forward** | ACTCACCTCTTCAGAACGAATTG |
|  | **Reverse** | CCATCTTTGGAAGGTTCAGGTTG |
| ACTB | **Forward** | CATGTACGTTGCTATCCAGGC |
|  | **Reverse** | CTCCTTAATGTCACGCACGAT |
| GAPDH | **Forward** | GGAGCGAGATCCCTCCAAAAT |
|  | **Reverse** | GGCTGTTGTCATACTTCTCATGG |
